# The Prognostic Ability of Log Odds of Positive Lymph Nodes in Oral Cavity Squamous Cell Carcinoma

**DOI:** 10.1097/MD.0000000000001069

**Published:** 2015-07-13

**Authors:** Ching-Chih Lee, Hsu-Chueh Ho, Yu-Chieh Su, Moon-Sing Lee, Shih-Kai Hung, Yen-Lin Chen

**Affiliations:** From the Department of Otolaryngology, Dalin Tzu Chi Hospital, Buddhist Tzu Chi Medical Foundation, Chiayi, Taiwan (C-CL, H-CH); School of Medicine, Tzu Chi University, Hualian, Taiwan (C-CL, H-CH, Y-CS, M-SL, S-KH); Division of Hematology-Oncology, Department of Internal Medicine, Dalin Tzu Chi Hospital, Buddhist Tzu Chi Medical Foundation, Chiayi, Taiwan (Y-CS); Department of Radiation Oncology, Dalin Tzu Chi Hospital, Buddhist Tzu Chi Medical Foundation, Chiayi, Taiwan (M-SL, S-KH); and Department of Pathology, Cardinal Tien Hospital, School of Medicine, Fu-Jen Catholic University, New Taipei City, Taiwan (Y-LC)

## Abstract

Recently, log odds of positive lymph nodes (LODDS) was proven a better prediction of outcomes than other methods in gastric cancer, pancreatic cancer, and colon cancer. However, the validity is not yet tested in oral cavity squamous cell carcinoma (OSCC). We conducted a retrospective study to compare the predictive ability of LODDS, traditional pN classification and lymph node ratio (rN) in OSCC patients.

In total, 347 OSCC patients receiving surgery with or without adjuvant therapy at the time of diagnosis between 2004 and 2013 were identified from the cancer registry database of the Dalin Tzu Chi Hospital. Cox proportional hazards models were used to compare the disease-specific survival (DSS) rates for pN, rN, and LODDS after adjusting for possible confounding risk factors. The discriminatory ability of different classification systems was evaluated using the adjusted hazard ratio and Akaike information criterion (AIC) by multivariate regression model. The prediction accuracy of the model was assessed by Harrell's c-statistic.

The 347 OSCC patients had a mean age of 57 years old. Among them, 322 patients (92.8%) were male and 189 patients (54.5%) were in stages III to IV. LODDS showed better discriminatory ability for patients with <5 pathological cervical metastatic nodes and those with rN < 0.2. The hypothetical T-LODDS-M staging system had higher linear trend Chi-square, lower AIC, and higher prediction accuracy compared with the American Joint Committee on Cancer (AJCC) TNM, or hypothetical T-rN-M system. After adjusting for other factors, the LODDS unfavorable group had the highest adjusted hazard ratio (HR, 5.42; 95% confidence interval [CI], 3.19–9.12) and LODDS-based model lowest AIC of 704, comparing with pN and rN-based model. The LODDS-based system had the highest prediction accuracy for 3-year DSS (Harrell's c-statistic, 0.803).

In our series, LODDS shows great promise as a prognostic tool for OSCC. Compared with the AJCC pN classification and the rN classification, LODDS can stratify OSCC patients and help to identify high-risk patients missed by the other systems.

## Abstract 2

Supplemental digital content is available in the text

## INTRODUCTION

Oral cavity squamous cell carcinoma (OSCC) is among the 10 most common forms of cancer, with a rising trend globally and in both Western and Asian countries.^1,2^ In Taiwan, the incidence of OSCC has continued to increase so that it is now the fourth most common cause of cancer-related mortality among men. Despite advances in clinical therapeutics, long-term survival of OSCC patients has improved little in the past several decades.^3,4^ A refinement in the present TNM Classification of Malignant Tumors (TNM) staging system may help better identify high-risk groups.

The present N (pN) classification of the American Joint Committee on Cancer (AJCC) staging system, which depends on the number and size of retrieved positive nodes, is primarily number based. The prognostic ability of pN may be influenced by the total number of lymph nodes removed and the pN classification requires a minimal number of retrieved nodes in order to prevent stage migration.^5–7^ The ratio-based system (rN), representing the ratio of positive nodes to total retrieved nodes, has been proven to better predict outcomes than pN.^6,8–10^ Our previous study validated the utility of rN utility in such major cancers as breast cancer, colorectal cancer, and HNC in Taiwan.^11^ Still, rN can better determine cancer prognosis than pN. Recently, log odds of positive lymph nodes (LODDS), which is calculated by the log of the ratio between the number of positive nodes and total retrieved nodes, has been utilized in only a few cancers such as gastric cancer, pancreatic cancer, and colon cancer.^5,12–14^ Compared with the pN or rN systems, LODDS has the unique strength of discrimination for cancer patients without positive lymph nodes, those designated as pN0 or rN0. Furthermore, LODDS can better discriminate between groups (eg, cancer patients with few positive nodes, subgroups with more homogeneity) even in gastric cancer patients with insufficient nodes retrieved.^5,15^

At present, there was no validation study about LODDS in head and neck cancer and the prognostic ability of LODDS for OSCC remains unanswered. The purpose of this study was to compare the ability of LODDS with pN and rN classification in predicting disease-specific survival (DSS) of OSCC patients.

## MATERIALS AND METHODS

### Ethics Statement

This study was approved by the Institutional Review Board of Buddhist Dalin Tzu Chi General Hospital in Taiwan. Review board requirements for written informed consent were waived because all personal identifying information was removed from the dataset before analysis.

### Database

The data for this study were collected from the Cancer Registry Dataset of the Buddhist Dalin Tzu Chi General Hospital Cancer Center from 2004 to 2013. The medical records and cancer registry dataset were retrospectively reviewed. Patients with newly diagnosed OSCC receiving radical surgery with or without adjuvant therapy were enrolled. Patients with distant metastasis at diagnosis, or those who underwent neoadjuvant chemotherapy or radiotherapy were excluded. The current series included 347 OSCC patients diagnosed between 2004 and 2013. Information in the cancer registry dataset includes the date of diagnosis, subsite of the primary tumor, age, gender, margin status (positive or negative), degree of differentiation (ie, well, moderate, or poor),^16^ total number of regional lymph nodes examined, number of positive regional lymph nodes,, presence of extra-capsular spread, chemotherapy regimen, radiotherapy regimen, cause of death, clinical TNM stage, and pathological TNM stage. All cases were staged according to the AJCC stage classification system, which was modified in 2009 (7th edition). The clinical endpoint was the DSS rate. Death from cancer was recorded as an event in our study and death from other causes was recorded as censored.

### Log Odds of Positive Lymph Node (LODDS)

LODDS was estimated using the calculation: log (pnod + 0.5)/(tnod − pnod + 0.5) in which pnod is the number of positive neck cervical lymph nodes and tnod is the total number of cervical lymph nodes retrieved.^17^

### Ratio-Based Lymph Node System (rN)

rN was derived from the number of positive regional lymph nodes examined divided by the total number of regional lymph nodes examined.

### The Optimal Cutoff Value for Lymph Nodes Classification

The optimal cutoff values for rN were determined as previous literature.^11^ The LODDS was determined by the following steps. We tried to select the 35%, 65%, and 85% cutoff point for the whole LODDS values. The final cutoff levels of LODDS were established as follows: LODDS0 (LODDS ≤ −1.58), LODDS1 (−1.58 < LODDS < −1.26), LODDS2 (−1.26 < LODDS < −0.82), and LODDS3 (−0.82 < LODDS). The cutoff value of rN was set as follows: rN0, 0; rN1, <0.2; rN2, >0.2 to <0.4; and rN3, >0.4, according to our previous research.^11^

### Statistical Analysis

All statistical operations were performed using SPSS (version 15, SPSS, Inc., Chicago, IL). Cumulative DSS rates for different N classifications (pN, rN, and LODDS) were analyzed using the Kaplan–Meier method and compared using the log-rank test. Survival curves were measured from the time of diagnosis using disease-specific mortality as the event variable. The prediction accuracy and discriminatory ability between the 3 staging system, AJCC TNM, hypothetical T-rN-M system, and hypothetical T-L(LODDS)-M system was assessed with Harrell's c-statistic and linear trend Chi-square test.^8,15^

The Cox proportional hazards regression model was used to compare outcomes of different N categories after adjusting for patient characteristics (age, gender, comorbid condition)^18^ and tumor status (differentiation and pathological T classification). In multivariate analysis, we merged the 4 classifications into favorable or unfavorable condition and compared the adjusted HR, Akaike information criterion (AIC), and Harrell's c-statistic for each regression model.^19,20^ Higher HR was taken to indicate a better system. In addition, smaller AIC was taken to indicate a more discriminatory system. We also used Harrell's c-statistics to describe the accuracy of prediction of the regression model or staging system as follows: 0.5 (equal to chance), 0.7 to 0.8 (acceptable), 0.8 to 0.9 (excellent), and 0.9 to 1 (outstanding prediction). A 2-sided *P*-value (*P* < 0.05) was considered significant.

## RESULTS

Table 1 shows the demographic data for these patients. This series consisted of 347 OSCC patients with a mean age of 56 years old. Among them, 322 (92.8%) patients were male and 189 patients (54.5%) were at an advanced pathological stage. The mean follow-up duration was 33 months. The overall 3-year DSS for the whole group was 76%. The mean number of total lymph nodes retrieved was 23.2 ± 13. The mean number of metastatic nodes was 1.04 ± 2.4. This series included 195 elective neck dissections for clinical lymph-node-negative OSCC patients and 152 neck dissections for clinical lymph-node-positive OSCC patients. One hundred forty OSCC patients (40.3%) with advanced pT classification, and most patients were with pN0 (67.7%) and pN2 (23.1%) (Table 2). The survival rates for 4 LODDS groups were summarized in Table 3. OSCC patients with higher LODDS incurred worse survival rates.

**TABLE 1:**
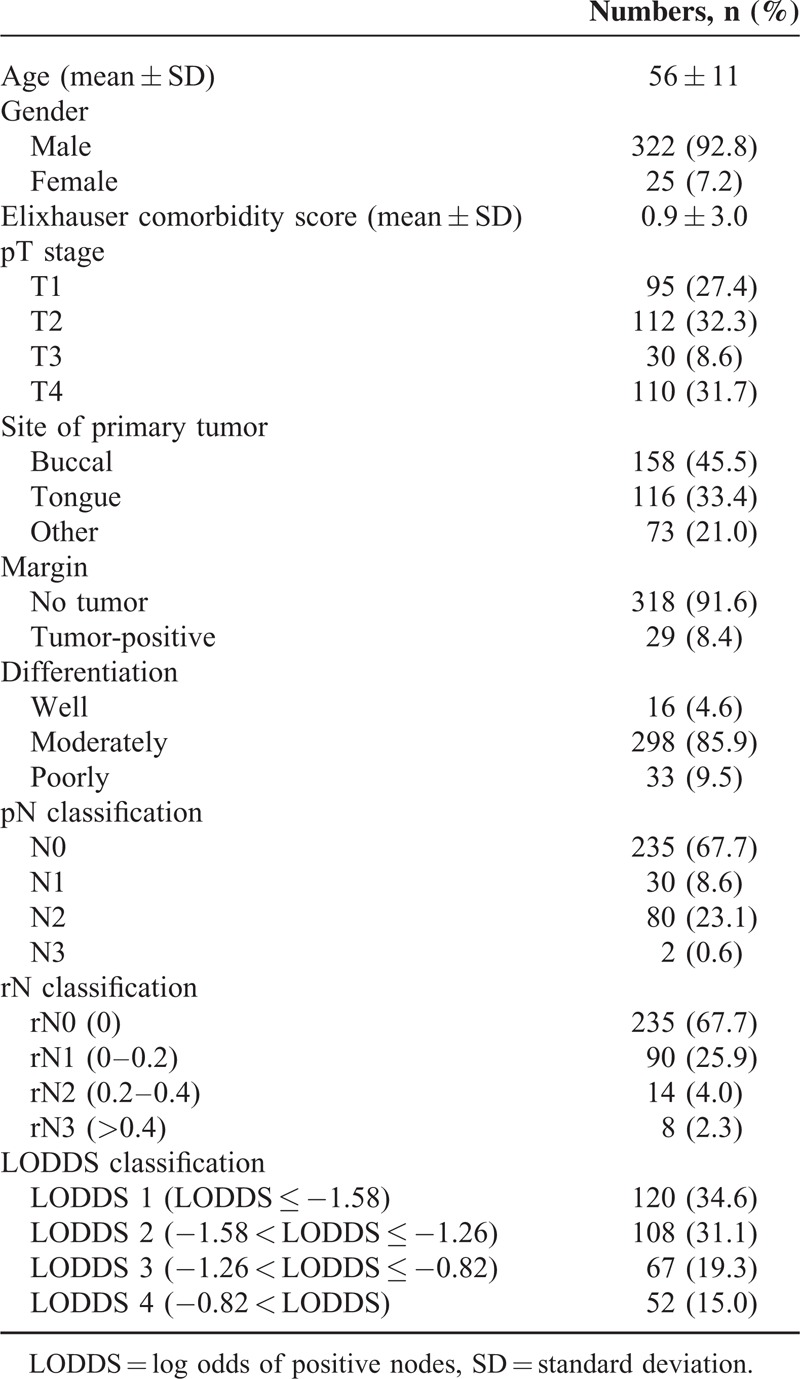
Demographic and Clinical Characteristics of Study Patients (n = 347)

**TABLE 2:**
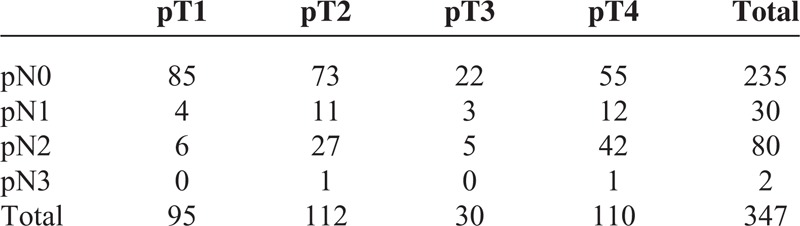
The Distribution of Pathological T and N Classification in Oral Cavity Squamous Cell Carcinoma Patients (n = 347)

**TABLE 3:**
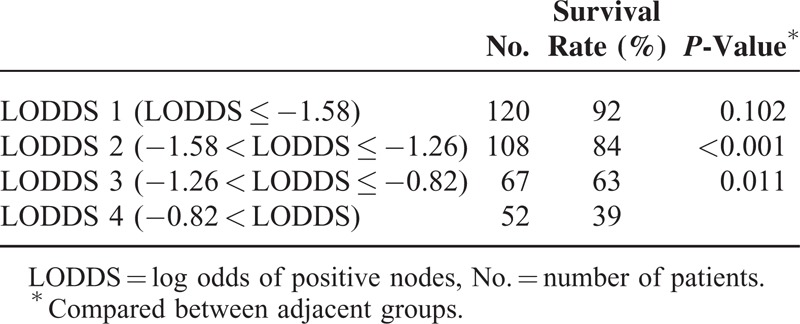
The 3-Year Disease-Specific Survival Rates of Oral Cavity Squamous Cell Carcinoma Patients According to the Value of LODDS

**FIGURE 1:**
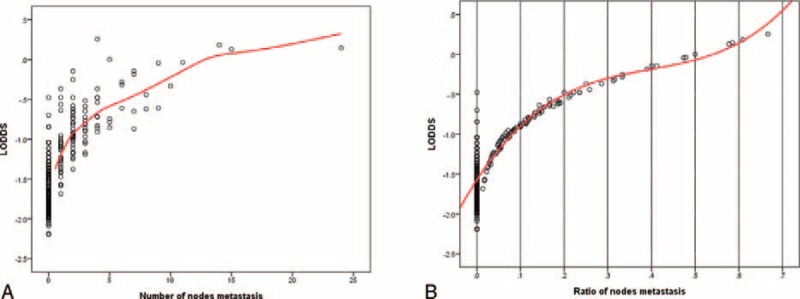
The distribution of LODDS, node metastasis, and rN.

Figure 1A shows the distribution of LODDS and the number of pathological positive nodes. The association was not linear. LODDS had better discrimination than pN for those with <5 neck metastases. Figure 1B demonstrates the association of LODDS and rN. This association was also nonlinear. LODDS had better discrimination than rN in HNC patients with rN <0.2 or >0.6. Furthermore, LODDS also demonstrated discriminatory ability for those with rN = 0. LODDS seemed to have better discriminatory ability than either pN or rN classification.

**FIGURE 2:**
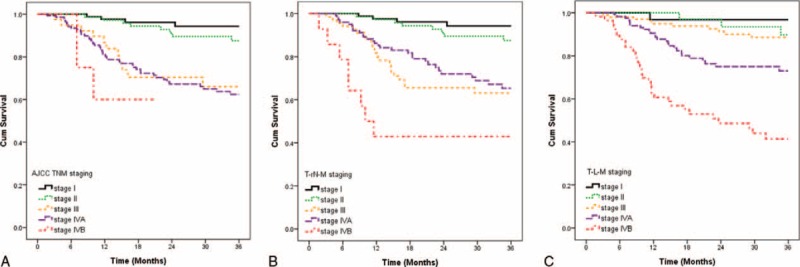
Impact of AJCC TNM (A), hypothetical T-rN-M (B), hypothetical T-L-M (C) staging on 3-year disease-specific survival in patients with oral cavity squamous cell carcinoma.

We examined the stage-specific survival rates (Figure 2). Table 4 summarized the performance between the AJCC TNM, T-rN-M, and T-L(LODDS)-M staging systems. The T-L-M staging system had higher discriminatory ability (liner trend Chi-square, 49; AIC, 739) and higher prediction ability (Harrell's c-statistic, 0.74) for 3-year DSS.

**TABLE 4:**
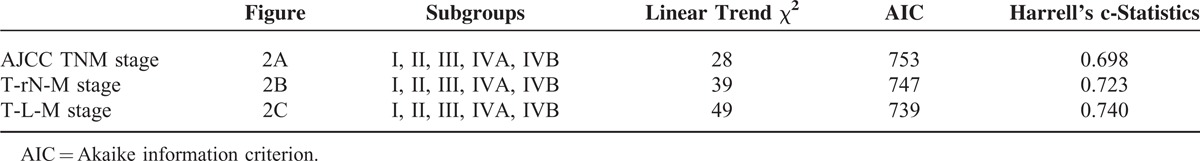
Comparison of the Performance of the AJCC TNM, Hypothetical T-rN-M, and Hypothetical T-L-M Staging system

**FIGURE 3:**
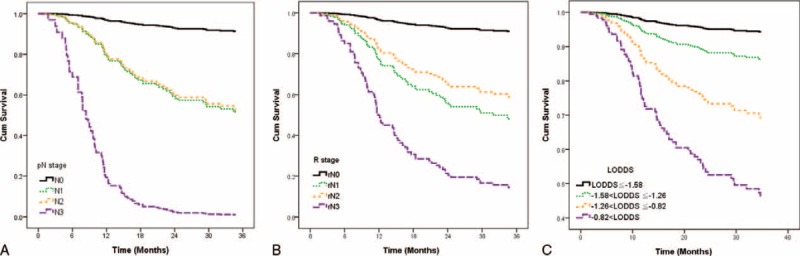
The adjusted disease-specific survival curves for pN, rN, and LODDS with 4 categories. After adjusting for age, gender, comorbidity, pathological T classification, margin status, differentiation, tumor site, there was inverse association between pN1 and pN2 in pN system (A), and rN1 and rN2 system (B). However, gradients of survival rate and LODDS were more reasonable and robust (C).

We used adjusted Kaplan–Meier survival curves to compare the discriminatory ability of the 3 systems, after adjusting for age, gender, comorbidity, pathological T classification, margin status, differentiation, and tumor site. The monotonicity of gradients was not well demonstrated in pN and rN classification (Figure 3A and B). OSCC patients with pN1 or rN1 had worse survival rates than those with pN2 or rN2. However, LODDS classification showed more reasonable and robust gradients of survival rates (Figure 3C).

**FIGURE 4:**
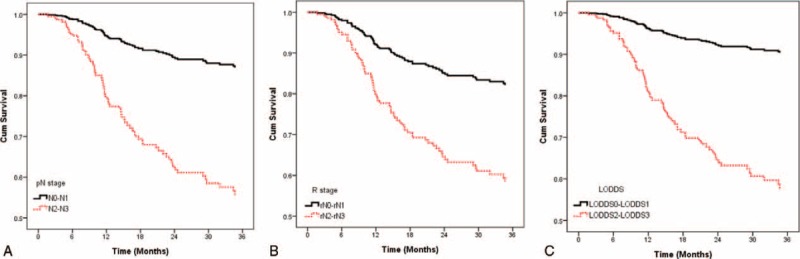
The adjusted disease-specific survival (DSS) curves for pN, rN, and LODDS with 2 categories. After adjusting for age, gender, comorbidity, pathological T classification, margin status, differentiation, tumor site, the difference in DSS between the favorable and unfavorable classification in LODDS system (C) (adjusted HR, 5.42) was the most significant, compared with pN (A) (adjusted HR, 4.19) and rN (B) (adjusted HR, 2.71) systems.

In order to make the model more stable, we merged the 4-category classification of cervical neck nodes into favorable and unfavorable (pN0–1 vs. pN2–3, rN0–1 vs. rN2–3, and LODDS 0–1 vs. LODDS 2–3, respectively). The adjusted DSS curves for the LODDS classification had better discrimination than the pN and rN classifications (Figure 4). In multivariate regression analysis, we compared the prognostic impact of pN, rN, and LODDS after adjusting for age, gender, comorbidity, pathological T classification, margin status, differentiation, and tumor site (Table 5). We used the adjusted HR and AIC to evaluate the discriminatory ability of each classification. LODDS had the highest adjusted HR (HR, 5.42; 95% CI, 3.19–9.12) and the LODDS-based model had lowest AIC value (704). The LODDS-based system had the highest prediction accuracy for 3-year DSS (Harrell's c-statistic, 0.803). The above-mentioned data indicated that LODDS is a superior classification system for OSCC compared to either pN or rN.

**TABLE 5:**
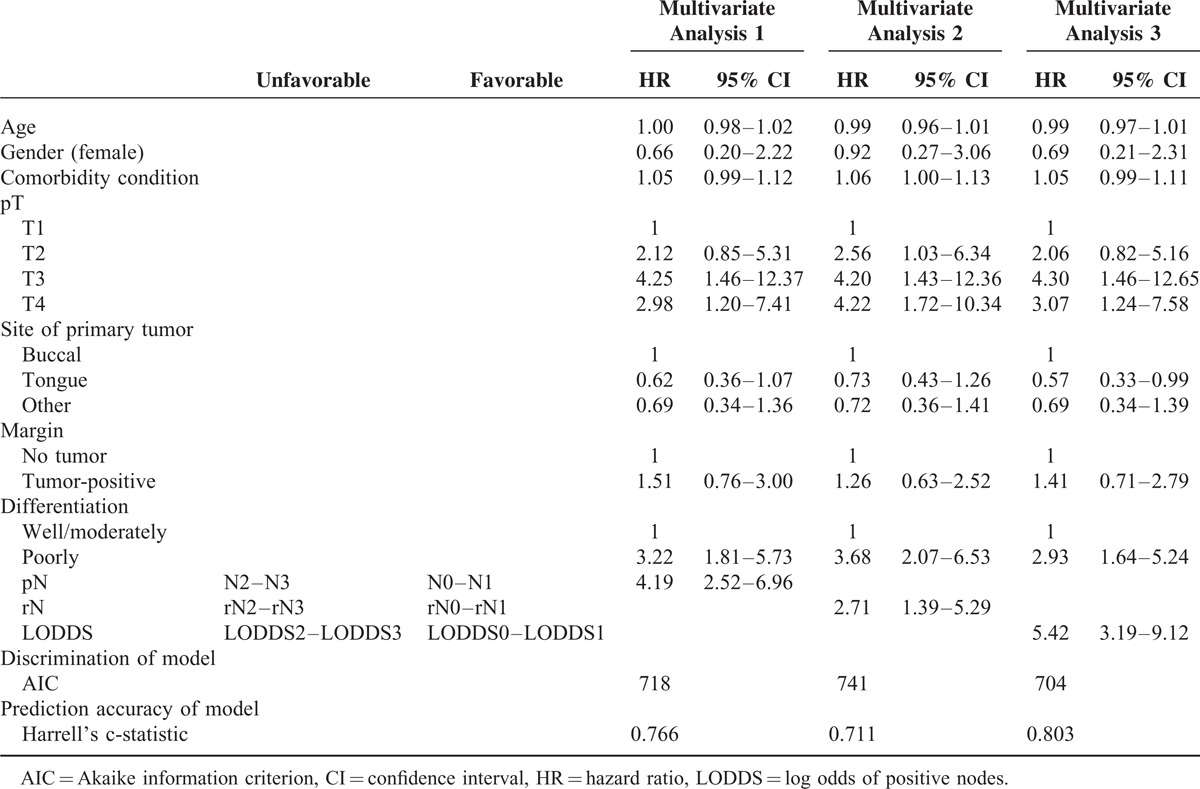
Multivariate Analysis of Overall Survival and Model Discrimination

## DISCUSSION

Our series validated the prognostic ability of LODDS classification for cervical neck lymph nodes in OSCC. Compared to pN or rN, LODDS had better discrimination for OSCC patients with <5 metastatic cervical nodes and had good discrimination for those with rN <0.2. The hypothetical T-LODDS-M staging system also provides better discriminatory ability and higher prediction accuracy, compared with AJCC TNM, or T-rN-M systems. LODDS had better homogeneity and monotonicity of gradients of survival rates after adjusting for other factors. LODDS may help us to stratify OSCC patients, especially those without pathological metastatic nodes or those without sufficient nodes retrieved.

Our series showed the superiority of LODDS to AJCC N classification, or rN in several perspectives. We tried to compare the hypothetical T-LODDS-M staging system with the AJCC TNM, and hypothetical T-rN-M systems in our series. The T-LODDS-M staging system had better performance with higher prediction accuracy (higher Harrell's c-statistic), and discriminatory ability (higher linear trend Chi-square). The T-LODDS-M system also had a smaller AIC, which represented optimal grouping and less loss of information in predicting mortality.^21^ In multivariate analysis, the LODDS incurred the highest HR and the model had the highest Harrell's c-statistic and lowest AIC, which implied better discriminatory ability and prediction accuracy.

**FIGURE 5:**
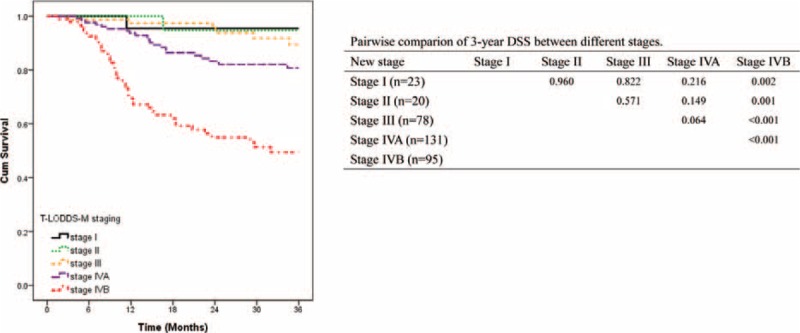
Three-year DSS based on hypothetical T-LODDS-M staging with cutoff points for LODDS as 25%, 50%, and 75%.

In order to construct a hypothetical T-L-M staging system, different cutoff points for LODDS was tested. First, 25%, 50%, 75% of LODDS value was chosen as cutoff points, and the 4 LODDS groups had significant impact on 3-year DSS in univariate and multivariate analysis (Supplementary Tables 1 and 2, http://links.lww.com/MD/A316) but this method would result in 226 (65.1%) patients with stage IV disease (Figure 5). Then we further selected 35%, 65%, and 85% as cutoff points and it grouped 179 (51.6%) patients as stage IV, which was a little closer to the AJCC TNM staging system which categorized 149 (42.9%) patients into stage IV in our series. However, the ideal cutoff points for LODDS may deserve a quantitative analysis for maximization of true positive rate and minimization of false positive rate in each LODDS category in the future.^5,15,22^

Regional lymph nodes metastasis is the most important prognostic indicator for outcomes in all patients with carcinoma, including HNC. Generally speaking, it is well known that cancer spreads from the primary tumor site to distant sites via the lymph nodes.^23^ Therefore, lymph node classification is considered one of the most important prognostic factors in cancer. For decades, N staging was used, based on a system of numbered lymph nodes. Recent focus has been on the total number of lymph nodes and the ratio of positive to negative lymph nodes.^13,24–28^ rN and LODDS are 2 new N classifications that are considered better than the traditional number-based classification system. An abundance of studies have reported the superiority of rN classification in various malignancies,^6,8,29^ but the recently developed LODDS has been little studied.

Some studies have found LODDS to be superior to pN and rN. Qiu et al^15^ compared LODDS and rN with pN (AJCC 7th edition), and concluded that LODDS is better in discrimination of gastric cancer prognosis. Similar results were seen by La Torre et al^13^ in pancreatic cancer. Another study of 440 colon cancer patients found that the overall survival rates of node-negative patients in the LODDS groups 0, 1, and 2 were 81%, 74.2%, and 50%, respectively (*P* = 0.020).^14^ In summary, conventional TNM staging pN and rN status cannot reliably classify between different groups of node-negative patients.

Two factors are believed to make LODDS classification superior to rN and pN classification. First, LODDS is able to discriminate among patients with the same ratio of node metastasis but different survival rates, as proposed by Sun et al.^5^ In addition, Wang et al^8^ considered that LODDS is a function of the number of negative lymph nodes, whereas LNR is a function of the total number of lymph nodes. The results from the present study showed a nonlinear association between the LODDS score distribution and the number of pathological-positive nodes. LODDS had better discrimination than pN for those with <5 neck metastases. The association of LODDS and rN was also nonlinear. LODDS had better discrimination than rN in HNC patients with rN <0.2 or >0.6. LODDS also showed discriminatory ability for those with rN = 0.

The primary flaw of the number-based UICC/AJCC pN classification is that the accuracy of the predicting prognosis is significantly influenced by the total number of nodes retrieved.^24,29–33^ The likelihood of identifying a positive node increases as more nodes are examined. However, it is virtually impossible to identify all the lymph nodes in the specimen. Herrera-Ornelas et al^34^ used a fat-dissolving technique to identify lymph nodes present within the specimen mesentery and found that 64% of the positive nodes were <5 mm in size. The ability to adequately recognize and accurately identify a positive lymph node remains an important issue. On the other hand, the ratio-based rN classification has been shown to be superior to pN in several solid malignancies including gastric cancer, lung cancer, breast cancer, colorectal cancer, and HNC.^27,35–37^ In our previous study, we found an association between poor prognosis and high rN in HNC.^11^ However, although the rN is a prognostic factor for HNC, the optimal cutoff value for rN seems to vary between studies. The flaws associated with traditional pN classification still exist, owing to the fact that rN0 classification is defined the same as pN0 classification. Finally, even though the rN classification has more power than pN to minimize the phenomenon of stage migration, retrieval of a minimum number of lymph nodes is still required to ensure its accuracy for prognostic assessment.^6^

Several limitations exist in the present study. First, we used Harrell's c-statistic, and AIC to evaluate the prediction accuracy and discriminatory ability in the model. Other procedures for internal validation of prediction models, such as split-sample, cross-validation, and bootstrapping could be considered.^38,39^ Second, although 347 OSCC patients were enrolled in the study, the number in each subgroup was relatively small. Third, we did not restrict the minimal number of retrieved lymph nodes in this analysis. In our series, 37 OSCC patients had <10 nodes retrieved. This may lead to stage migration in pN classification.^13,24,29,32,40^ Fourth, although LODDS demonstrated better discrimination than pN in those with <5 metastatic cervical nodes and better than rN in those with rN <0.2, the lack of events prevented subgroup analysis of the 3 classification systems. Large-scale prospective studies or those using a population-based cancer registry database may overcome these limitations. Our series consisted of 92% male OSCC patients, who were mainly attributed by betel-nut chewing, alcohol, and smoking among men in Taiwan and validation of the above-mentioned findings in cohorts among the Western countries may help us to generalize the applicability of LODDS in OSCC.^41,42^

## CONCLUSION

In our series, LODDS shows great promise as a prognostic tool for OSCC. LODDS >−1.26 in head and neck cancer was negatively associated with DSS after adjusting for other factors. Compared with the AJCC pN classification and the rN classification, LODDS can better stratify OSCC patients and help to identify high-risk patients missed by the other systems.

## Acknowledgment

All authors thank the staff in the Center for Clinical Epidemiology and Biostatistics for data preparation.
